# Paradoxical Duel Role of Collagen in Rheumatoid Arthritis: Cause of Inflammation and Treatment

**DOI:** 10.3390/bioengineering9070321

**Published:** 2022-07-15

**Authors:** Jeevithan Elango, Camilo Zamora-Ledezma, Baolin Ge, Chunyu Hou, Zhilin Pan, Bin Bao, Carlos Pérez Albacete Martínez, José Manuel Granero Marín, José Eduardo Maté Sánchez de Val, Chunling Bao, Wenhui Wu

**Affiliations:** 1 Department of Biomaterials Engineering, Faculty of Health Sciences, UCAM-Universidad Católica San Antonio de Murcia, Guadalupe, 30107 Murcia, Spain; srijeevithan@gmail.com (J.E.); jemate@ucam.edu (J.E.M.S.d.V.); 2 Department of Marine Bio-Pharmacology, College of Food Science and Technology, Shanghai Ocean University, Shanghai 201306, China; 13561936983@163.com (B.G.); ytxiaoyu@163.com (C.H.); zlpan4869@163.com (Z.P.); bbao@shou.edu.cn (B.B.); 3 Tissue Regeneration and Repair Group, Biomaterials and Tissue Engineering, Faculty of Health Sciences, UCAM-Universidad Católica San Antonio de Murcia, Guadalupe, 30107 Murcia, Spain; czamora9@ucam.edu; 4 Oral Surgery and Oral Implantology Department, UCAM-Universidad Católica San Antonio de Murcia, 30107 Murcia, Spain; cperezalbacete@ucam.edu; 5 Department of Implant Dentistry, Faculty of Medicine and Dentistry, UCAM-Universidad Católica San Antonio de Murcia, 30107 Murcia, Spain; jmgranero@ucam.edu; 6 The Sixth People’s Hospital Affiliated to Shanghai Jiaotong University School of Medicine, Shanghai 201306, China

**Keywords:** collagen, collagen peptide, arthritis, joint treatment, mechanism, homeostasis

## Abstract

In biology, collagen-biomaterial regulates several signaling mechanisms of bone and immune cells involved in tissue repair and any imbalance in collagen turnover may affect the homeostasis of cells, becoming a major cause of several complications. In this case, the administration of oral collagen may play a potential role in returning cells to their normal function. For several decades, the beneficial effects of collagen have been explored widely, and thus many commercial products are available in cosmetics, food, and biomedical fields. For instance, collagen-based-products have been widely used to treat the complications of cartilage-related-disorders. Many researchers are reporting the anti-arthritogenic properties of collagen-based materials. In contrast, collagen, especially type-II collagen (CII), has been widely used to induce arthritis by immunization in an animal-model with or without adjuvants, and the potentially immunogenic-properties of collagen have been continuously reported for a long time. Additionally, the immune tolerance of collagen is mainly regulated by the T-lymphocytes and B-cells. This controversial hypothesis is getting more and more evidence nowadays from both sides to support its mechanism. Therefore, this review links the gap between the arthritogenic and anti-arthritogenic effects of collagen and explored the actual mechanism to understand the fundamental concept of collagen in arthritis. Accordingly, this review opens-up several unrevealed scientific knots of collagen and arthritis and helps the researchers understand the potential use of collagen in therapeutic applications.

## 1. Introduction

Cartilage is a connective tissue, mainly composed of an extracellular matrix, cartilage cells, and fibers without blood vessels and nerves, and thus gets nourished through perichondrium from blood vessels by the permeation of nutrients into the intercellular substance. The major cells of cartilage, chondrocytes regulate the dynamic stability and homeostasis of cartilage during anabolism and catabolism [1,2]. Any disturbance in this balance may lead to damage in the cartilage tissue, leading to arthritis, which could be due to several internal and external factors such as inflammatory environments (including but not limited to cytokines such as interleukin (IL)-1β, tumor necrosis factor (TNF)-α, and IL-6 that potentially trigger the cellular immune system. The cytokines are the major factors to stimulate the matrix metalloproteinases (MMPs) and aggrecanase (ADAMTS-4 and 5) production, leading to the progression of arthritis, a common arthritis due to damaging the articular cartilage membrane. NF-kB pathway is essential to represent the link between IL/TNF-α cytokines in order to stimulate MMPs and SRY-related high-mobility group-box gene 9 (Sox9) [3,4]. IL-1β is mainly secreted by the immune cells such as macrophages, natural killer (NK) cells, dendritic cells (DCs), neutrophils, T lymphocytes, and B lymphocytes [5]. It was reported that IL-1β can downregulate the expression of chondrocyte-specific genes Sox9 and collagen type II α (COL2A1), which is potentially accelerated by T lymphocytes secreted IL-3 [6,7]. On the other hand, IL-3 and IL-6 produced by T lymphocytes protect the cartilage by inhibiting MMPs induction through tissue inhibitors. In some cases, IL-6 inhibits proteoglycans synthesis in chondrocytes and elevates IL-1β for proteoglycan denaturation. Accordingly, IL-6 has a dual role in protecting and destructing effects on cartilage cells, which are purely influenced by the response of the immune system against allergens [8]. For its part, transforming growth factor β (TGF-β) is one of the major cytokine produced by T cells compared to other body cells, which have a positive effect on cartilage remodeling by upregulating differentiation-related transcription gene Sox9 and downregulating IL-6 receptor expression in chondrocytes, thereby maintaining cartilage homeostasis [9,10].

Rheumatoid arthritis (RA), a well-known autoimmune disorder due to the imbalance of the microenvironment around joints and cartilages, is an inflammatory synovial disease that potentially damages cartilage matrix such as collagen (type II, IX, and XI), hydroxyapatite, and proteoglycans due to age-related disorder or other external and internal factors. This could be possible by stimulating auto-immunity through self-antigen, where immune cells like B and T cells get activated, recognize their own antigen through major histocompatibility complex (MHC), follow-up the destruction of their own tissues, and downregulate the regeneration process of tissues, a major cause of arthritis. For instance, the antibodies against type II collagen (CII) play a major role in creating a situation in which a cartilage tissue does not regenerate. In the case of B and T cells present in rheumatoid synovial fluid and synovium, the higher level of CII-induced antibodies is elevated [11,12,13,14,15,16]. Many antigens such as viral proteins from Cytomegalovirus and Epstein-Barr virus and autologous proteins released from diarthrodial joints are involved in the activation of immune cells, especially T cells that can damage the cartilages leading to arthritis [17,18]. One of the familiar autologous proteins is CII, a potential antigen for RA, due to the evidence that it triggers autoimmunity in synovium and cartilages of diseased animal models [13,19,20,21,22,23,24]. Therefore, it is evidenced that the induction of both B and T cells by CII is a critical process for collagen-induced arthritis (CIA) in rodents [25] and humans [26].

Surprisingly, a study reported a controversial finding of collagen and arthritis hypothesis: intravenous injection of CII could potentially suppress a CIA model by increasing a crucial anti-inflammatory mediator of CIA, IL-4 [27]. However, the exact mechanism behind this effect was not justified by this study. To support this finding, several in vitro, in vivo, and clinical studies claimed the anti-arthritic effect of collagen and peptides. For instance, a study published in Science Advances by Katsumata et al. revealed that collagen bound anti-TNF-α antibody by conjugating a collagen-binding peptides (CBP) (LRELHLNNNC) derived from the sequence of decorin suppressed arthritis development after accumulation in the inflamed paw of the arthritis model [28]. In another study, Devasia et al. compared the anti-arthritis effect of bioactive collagen dipeptide (containing high proline, hydroxyproline, and hydroxyproline glycine) with conventional collagen peptide and stated that bioactive collagen peptide at 517 mg/kg suppressed arthritis induced by monoiodo acetic acid [29]. In addition, Hakuta et al. claimed that the treatment of collagen tripeptide (Gly-X-Y tripeptides) could reduce the inflammation (chemokine and type 2 cytokine production from keratinocytes) through STAT1 phosphorylation [30]. The structural variations such as single chain and triple-helix of collagen and its peptides regulate the biological properties. Though collagen had been reported to have many beneficial effects, several researchers constantly report solid evidence to prove the inflammatory and arthritogenic effects of collagen. Moreover, the CIA model has been widely recognized as a standard procedure to mimic RA, since they share a similar pathology. In this case, arthritis is generally induced by oral immunization of CII doses with or without adjuvants. The actual mechanism of CII in arthritogenic and immunogenic induction has been elaborated below in Section 2. Therefore, we tried to explore the actual theory of whether collagen does support or not in the case of arthritis treatment, and we perceived contradictory findings from the available literature. As biomaterial scientists, especially those working with collagen biomaterial, we should understand this fundamental concept in order to convey reliable evidence of the beneficial effect of collagen in biomedical applications. To this end, we intend to summarize the actual mechanism of collagen in arthritis based on the available evidence from the literature in this review.

## 2. Mechanism of Collagen in Arthritis Induction

### 2.1. Interaction of Collagen with T Cells

We have collected the data for the possible cellular mechanism to show the induction of T cell-dependent immunity by collagen in arthritis. In general, many studies claimed that the CII glycosylated peptides 256–270 are the major immunodominant T-cell epitopes involved in CIA. As evidence, the transgenic mice model expressed with immunodominant CII glycosylated 256–270 epitope in cartilage had partially tolerated T cells, which crucially develop CIA. T cells are formed from multipotent stem cells in the bone marrow and are transported into the thymus for maturation by positive and negative selection, which is approximately completed in two weeks [31,32]. Then, mature (or naïve) cells are circulated to hire a peptide-MHC antigenic complex in order to activate the immune system [33].

**Figure 1:**
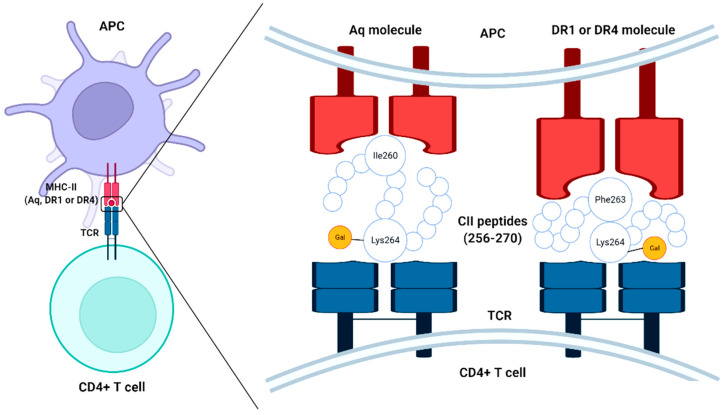
Schematic representation of the chimerism of type II collagen (CII) peptides (256–270) to MHC-II molecules and T cell receptor (TCR) of CD4 + T cells, APC-antigen-presenting cell.

The role of CII-derived peptides combined with the MHC class II molecules is apparent in the development of arthritis in the CIA model [34,35,36]. Earlier, the shared epitope hypothesis of MHC class II molecule study revealed that group of related epitopes (Dw4, Dwl0, Dw13, Dw14, and Dw15) found in many of the human leukocyte antigen (HLA)-DR genes (HLA-DR1 and HLA-DR4) can confer disease risk [37,38]. I-Aq is a major MHC class II allele for specific protein binding that has been identified in the CIA model shares a similar peptide-binding pocket of epitope expressed in DR1 and DR4 molecules (DRB1*0401 and DRB1*0101) derived from position 260–273 of the CII molecule (Figure 1). More exactly, the Aq molecule binds with peptide via isoleucine 260 in the P1 pocket, whereas DR4 and DR1 molecules bind with phenylalanine 263 in the P1 pocket. However, the possibility of arthritis induction by collagen alone is still under argument, since the incidence of arthritis can be possible even without cartilage-specific autoimmunity in various adjuvant-induced arthritis models [39,40] and transgenic animals with deviant resistance to ubiquitously expressed proteins [41,42,43]. To support this hypothesis, several studies claimed that autoreactive T cells specific for CII were not identified from either RA joints or the CIA model [44,45], which was further corroborated by another study using DR4 + CII peptide tetramer [46].

Likewise, the MHC class II molecule has a major role in arthritis induction; for instance, the Aq molecule expressed by the H-2q haplotypes and DRB1*0401/DRA molecule in the DR4 haplotype is associated with CIA in mice and RA in human, respectively [37,47]. The association of MHC molecule has the structural relevance since peptide binding cleft of the DRB1*0401/DRA and Aq molecule are closely relative and attach to the similar CII peptides region [48,49].

Therefore, the CIA model is very useful to mimic the RA microenvironment and investigate the possible interaction of cartilages with immune cells. CII present in cartilages of different animals might not have to vary distinguished amino acid composition, for instance, the triple-helical region of CII in mice had substituted only 13 out of 1015 amino acids compared to rats [50,51], which even might be due to the extensive post-translational modifications such as glycosylation of hydroxylysines and hydroxylation of lysines and prolines. It is stated that immune response is likely regulated by the modification in two binding epitopes for glycosylations and three sites for hydroxylations [35].

To understand the occurrence of arthritis by tolerized T cells or naïve T cells, as the theory speculates, the interaction of non-tolerized T cells rather than tolerized T cells could be the major reason for the immunogenicity of CII; Corthay et al. [52] developed a thymectomized adult mice model immunized with CII for 4 weeks in order to tolerate the T cells and found the occurrence of arthritis, which could be due to IFN-γ production by partially tolerant T cells with the help of B-cell though the existence of peripheral tolerance against the glycosylated CII 256–270 epitope. The occurrence of arthritis by T cells depends on various factors such as the nature and amount of degraded CII moved to the lymph node (the place where CII is supposed to meet T cells), MHC binding ability, and the nature of antigen-presenting cells (APCs) transport. It is still questionable how the CII released from cartilage is introduced to peripheral circulation because there is no lymphatic drainage in cartilage. It is likely, but not proven, that the proteins scavenged from cartilage are engulfed by numerous macrophages surrounding synovial tissue and transported to lymph nodes, thereby reacting with the immune system, whereas DCs are not involved in this process [53,54]. Therefore, the appearance of CII in the periphery is inadequate; however, it must take place.

One of the possible explanations for this contradictory debate might be the specific tolerance induced in both animals and humans. For instance, mice expressed with the heterologous form of immunodominant CII 256–270 epitope in cartilage had partial tolerance against T cells, which prolongs the induction of CIA. However, these animals induced arthritis upon treatment of effectors such as IFN-γ by assisting B cells to produce anti-CII IgG antibodies [55]. In this case, the induction of arthritis could be due to the interaction of non-tolerized newly formed T cells from the thymus with CII during immunization.

In general, the immune system recognizes the self-antigen regular basis, but the immune system tolerated this self-antigen, thereby triggering no inflammation. This is why the tolerance ensues against cartilage-derived collagen peptides expressed on APCs in peripheral lymphoid organs; however, the effect is reversible upon the breakage of tolerance by external factors. Hence, the T cells that are specific for CII seem normally to ignore the existence of the self-antigen unless that is activated by any other means. Moreover, the immune response for self CII is more likely immutable compared to external CII due to the existence of established tolerance to cartilage-derived CII 256–270 peptide. In the case of the CIA model in mice, we speculate that the immune system recognizes the external CII and peptides, which might have unique peptide sequences (epitopes) as a foreign antigen, which triggers adverse effects by immune cells, though they are tolerized against self CII on its own. In other cases, it is also justified that the breakage of tolerance and hindering T cells possibly happened by the introduction of adjuvant, which potentially activates APCs for the inflammation. However, at present very little is known about the possible causative reason for the breakage of tolerance in this case.

Collagen peptides taken as food supplements are converted into oligopeptides containing glycine and hydroxyproline as major amino acids that are circulated in peripheral blood and reported to have beneficial effects. The actual mechanism of these collagen oligopeptides in the immune system still needs to be explored. The earlier study disclosed the role of collagen peptides (Pro-Hyp and Hyp-Gly) on immune cells differentiation and concluded that serum OVA-specific immunoglobulin E (IgE) production and anaphylaxis responses were downregulated by collagen peptides [56]. This could be achieved by regulating T-helper (Th) type 1, and (Foxp3+) regulatory T (Treg) cells differentiation by upregulating IFN-γ-induced signal transducer and activator of transcription 1 (STAT1) signaling either in the presence or absence of TGF-β. Finally, they concluded that collagen-peptide downregulates inflammation responses by accelerating the CD4 + T cells’ growth toward Th1 and Treg cells, through enhancing IFN-γ-mediated STAT1 phosphorylation in pre-activated CD4 + T cells followed by Janus kinase (JAK)-STAT signaling, which is supposed to participate in reducing immune responses mediated by Th2 [56]. To support this finding, Th1-associated cytokine, IFN-γ is believed to obstruct the expansion of Th2 pathologies, which potentially participate in hypersensitivity reactions by activating IgE production to antigens through Th2-associated cytokines such as interleukin (IL)-4, -5, and -15 [57]. Other studies claimed that Treg cells participate in maintaining healthy immune responses to allergens by downregulating numerous immune responses such as antigen-specific Th cells and innate effector cells proliferation via Th1- and Th2-associated cytokines production [58,59]. When administered orally, the collagen peptides (Pro-Hyp and Hyp-Gly) were absorbed in local inflammatory sites in a murine contact-dermatitis model and take part in inflammation and wound healing by regulating the chemotactic action of fibroblasts [60], monocytes [61] and neutrophils [62]. It has also been established that consumption of collagen peptides advances human immunological status as evaluated by 14 immunological parameters, proposing that collagen peptides suppress immune functions and responses [63].

An earlier in vivo study model using T cell hybridomas derived from C3H.Q and DBA/1 mice immunized with rat CII confirmed the specificity of T cell hybridomas for the CII (256–270) segment. This specific recognization pattern of T cell determinants was altered by the posttranslational modifications (hydroxylation and variable O-linked glycosylation) of the lysine at position 264, which is precisely recognized by different T cell hybridoma subsets. From the T cell receptor (TCR) sequencing, they have confirmed that each T cell epitopes selected its own TCR repertoire and T cell hybridomas (20/29) specifically recognized CII (256–270) glycosylated with a monosaccharide (D-galactopyranose).

The recognition of CII by T cells is a crucial step in the stimulation and growth of arthritis and thus the determinants of T cells with CII play a keen interest in this aspect [64,65]. A possible scenario from the available literature claims that T cells can bind with CII in a unique mechanism, more specifically through carbohydrates residues (O-linked carbohydrate side chains to one or two saccharides of CII) and specific amino acid residues (256–270) pattern of CII [66]. Recent studies reported that the T cell contact with CII (256–270) could be achieved through lysine at position 264 anchoring at Aq molecule with I260 and F263 [67,68], which confirms the importance of lysine side chain modifications in T cell recognition.

Accordingly, CII (260–267) and CII’s proteolytic fragments were found to be the core of the immunodominant peptides [36,69], and arthritogenic, respectively, although less so than native CII [36]. The affinity of I-A^q^ (MHC class II allele) molecule with CII could be achieved by the two amino acid residues such as phenylalanine at position 263 (Phe263) and isoleucine at position 260 (Ile260) of the CII peptide as binding anchors to the I-A^q^ molecule [69,70]. Similarly, DR1 and DR4 anchor determinant core CII (263–270), more specifically at Phe263 and Lys264 for the presentation of the peptide and binding affinity, is much higher in DR1 than in DR4 [71].

### 2.2. Interaction of Collagen with B Cell

To date, the direct role of T cells in CIA pathogenesis is not substantially established. Indeed, the response of B cells through anti-CII immunoglobulin is predominantly by Immunoglobulin G2 (IgG2) to trigger a complementary cascade, which is a vital phase for the development of CIA. Antibodies against CII are important in the development of CIA and possibly also in RA.

**Figure 2:**
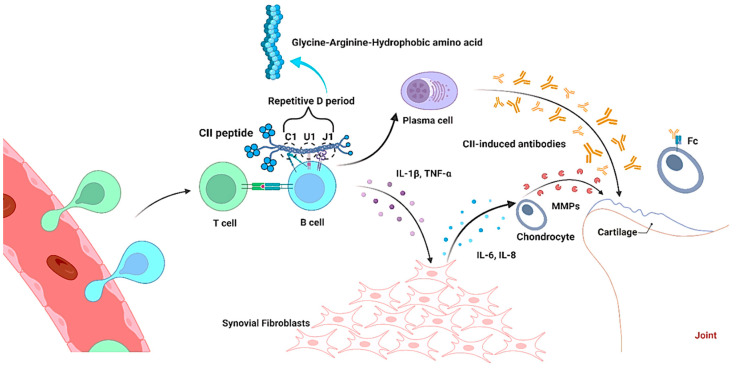
Interaction of type II collagen peptide with B cell in collagen-induced arthritis (CIA) pathogenesis. Collagen with Gly-Arg and hydrophobic amino acids interacts with B cells through repetitive D period (C1, U1 and J1), which releases cytokines and CII-induced antibodies in order to damage the joints during arthritis.

The role of B lymphocytes in the immune system is well known, which is derived from bone marrow as pluripotent stem cells that take five stages to get fully mature cells such as pre-B lymphocytes, immature B lymphocytes, mature B lymphocytes, activated B lymphocytes, and plasma cells. Interestingly, B lymphocytes can easily penetrate the synovium and damage the cartilages during the occurrence of arthritis. The movement of B lymphocytes into the synovium is highly dependent on the presence of plasma cells, lymphoid follicles, and the inflammation of the synovium [72]. It was stated that the cytokines such as TNF-α, IL-1β, IL-6, IL-8, and IL-18 secreted by B lymphocytes and synovial fibroblasts (SFs) potentially regulate the balance of anti-inflammatory and pro-inflammatory factors during arthritis [73,74,75,76,77]. The possible mechanism of B lymphocytes on cartilage damage was reported by Störch et al. [78]. The TNF-α secreted by B lymphocytes is a crucial mediator for SFs activation in joints, and further activates MMPs secretion through the production of cytokines such as IL-6 and IL-8 by SFs, thereby destruct the cartilage tissues (Figure 2).

Studies claim that administration of CII-induced monoclonal antibodies (mAbs) bind cartilages through the Fc receptor and induce severe arthritis participating in the infiltration of macrophages and neutrophils [79,80,81,82]. The production of antibodies against CII is completely dependent on the structural features of collagen, and therefore several studies initiated to identify the specific epitopes in CII using mice and rats models [83]. These studies confirmed that the major epitopes on triple-helical CII such as C1, U1, and J1 share the common motif comprising repetitive glycine-arginine-hydrophobic amino acid residues on the cartilage [84], and they share a similar immunodominance with animals (mice and rats) and humans [16,83,85], as well as have the strong potential arthritogenic ability, whereas F4 epitope does not [16].

Earlier studies conducted with B cell-deficient mice confirmed the solid evidence of arthritogenic efficiency of B cells triggered by CII [86,87]. It is evidenced that the response of B cells to CII depends on engaging the germinal center of B cells through T cells, which principally trigger isotype to IgG following antibody production against CII. It is also clear that the recognization pattern of the CII by B and T cells for immunization differs from that of CII derived endogenously from cartilages, which potentially alters the specificity of the antibody response to CII. It is evidenced that mice expressing Aq molecule of MHC class II recognize the similar epitopes of T and B cells and the affinity of rat CII peptide reorganization with T cell is more pronounced compared to mouse CII peptide [70]. It has been reported that the reorganization pattern of T cells with CII peptide for immunization depends on the molecular and glycosylation pattern of CII [88,89].

In general, the induction of arthritis is triggered by collagen-induced antibodies with lipopolysaccharide boosting injection [78,79,90]. It has been reported that exposure to modified CII from cartilage triggers adaptive immunity to CII in some patients, though immunization of priming is not sufficient to cause RA in rats and mice [91].

Notably, the induction of immunization with native CII (intact triple-helical structure) is essential during CIA induction [21,92]. The triggering of functional B cells by the conformation changes of CII is crucial to the development of CIA pathogenesis [87,93]. Earlier in vivo studies with transgenic mice DBA/1 (H2q), which lack negative selection of CII autoreactive B cells, showed that the early activation of IgG secreting autoreactive B cells upon immunization with heterologous rat CII could recognize immunodominant native structures on the triple-helical moiety of the autologous CII molecule [87,94]; however, they do not cross-react with any of the systemically available collagens like type I collagen (CI) regardless a homology of 80% at the amino acid level. After intraperitoneal injection into syngeneic mice, the CII-specific autoantibodies (activated IgG) induce synovial inflammation and even erosive arthritis by entering articular cartilage [79,93,95,96].

Studies on characterization and mapping of the arthritogenic epitopes of CII exposed several linear epitopes covering the entire sequence of the immunodominant CNBr-fragment 11 (α1(II)CB11) could be responsible for B cell immunity and disease [97,98]. Extensively, the collagen microfibril (repetitive D period of 66 nm length) is possibly subdivided into three segments based on its size (at a ratio of approximately 2:1:2) by the three loci of B cell epitope clusters such as E/F10/J1, C1I-III, and D3/F4. In a native environment of intact cartilage, the heterotypic interactions of CII with other matrix molecules such as decorin [99], fibromodulin [100], collagen XI [101], and collagen IX [102,103] produce more complex structured microfibril, which potentially constrains the accessibility of collagen to the recognition of antibody, due to masking of most of the collagen surface (heterotypic fibrils) by other extracellular matrix molecules. In the initial phase of CIA, B cell epitopes are less densely attached to CII molecules, and the potential localization of B cell epitopes with collagen fibril materializes through reconstruction of collagen IX and XI molecular arrangement to CII via altering dimensions of the rigid triple-helical molecules and localizations of intermolecular cross-links [101,102,103]. It has been reported that the localization of B cell epitopes can occur within the less densely packed gap region (C1I-III), the borders of the gap region (E/F10I + II/J1 and D3/F4), and the clusters formed through merging the non-collagenous domains (three of four) of collagen IX and the telopeptide regions of collagen II and XI with the same regions of the D period on CII [85].

The R-G hydrophobic motif contained dominant epitopes, and this is reminiscent of the organization of B cell epitope. B cells are potentially activated by the contact of antigen through optimal Ig receptor cross-linking [104]. The possible mechanism of B cells in arthritis induction was elucidated by Schulte et al. [85]. Immunoglobulin V-gene (light-chain region) selected B cells of arthritis-prone DBA/1 mice showed higher expression of anti-CII IgG by interacting with repetitive determinants in discrete accessible sites of collagen fibrils in cartilage, which resulted in positive selection rather than deletion of autoreactive B cells. During the cartilage resorption in endochondral bone formation, the B cells can meet with CII in the bone marrow and moved to the peripheral circulation, where they may encounter cognate T cells assistance upon antigen exposure. All these sequences of events create a pre-actuated group of clonally designated CII-specific B cells and they are recruited by the arthritogenic immune system upon CII immunization for the destruction of cartilage either by producing anti-CII IgG or lowering the threshold of autoreactive T-cell engagement in order to endorse the presentation of antigen to activated B cells [60,61]. Although, it is important to note that the activation of B cells against CII is directed by immunization by requirements of T cells. The preimmune response by the accessibility of CII determinants in the intact cartilage tissue allows the autoantibodies to bind intact cartilage in vivo in order to prove CII as an immunoprivileged self-protein.

Intriguing evidence from the K/BxN arthritis transgenic mice model indicated that the TCR plays a crucial role in the development of joint disorders, especially in RA [105]. More substantial evidence concerning the pathogenicity of B cells in RA was recently obtained from human clinical trials with a refractory disease by using B-cell depletion with a human chimeric anti-CD20 monoclonal antibody rituximab (Rituxan) [106,107]. Even though the ablation of pathogenic B-cells and their precursors had a beneficial effect on autoimmune disorders, antibody-secreting plasma cells are the potential source of autoimmune disorders induction [108,109]. Therefore, not only antibody production, but the other functions of B cells such as antigen presentation, cytokine production, and provision of costimulatory signals to T cells might also play vital roles in disease pathogenesis [110].

Few studies proved that deletion of mutation of IFN-γ in the B6 IFN-γ-KO CIA model highlighted the pathogenic role of B cells in arthritis development; CBA/N xid43 mice (defective B cell development by X-linked immunodeficiency) and muMT mice (depletion of B cells by deletion of the IgM heavy chain gene) did not produce antibody response at the time of CII immunization; they were therefore resistant to CIA induction [86,96]. B10.Q(BALB/c × B10.Q)F2 mice model showed that the response of B cells predominantly depends on the specific CII’s triple-helical peptides of the major B cell epitopes such as C1, U1, and J1 upon chronic arthritis induction. Thus, the development of chronic arthritis is purely related to an arthritogenic specific antibody response to certain epitopes of CII. This effect was further verified by treating animals with denatured CII α-chains that produce only weak antibody responses without arthritogenic signs [111]. This evidence substantiated that the native or synthetic triple-helical peptides corresponding to arthritogenic epitopes (C1, U1 and J1) are essential to trigger B cells response in order to establish severe and chronic arthritis [16,82].

## 3. Collagen Treatment Strategy in Arthritis

### 3.1. Collagen Supports for Arthritis Treatment

**Figure 3:**
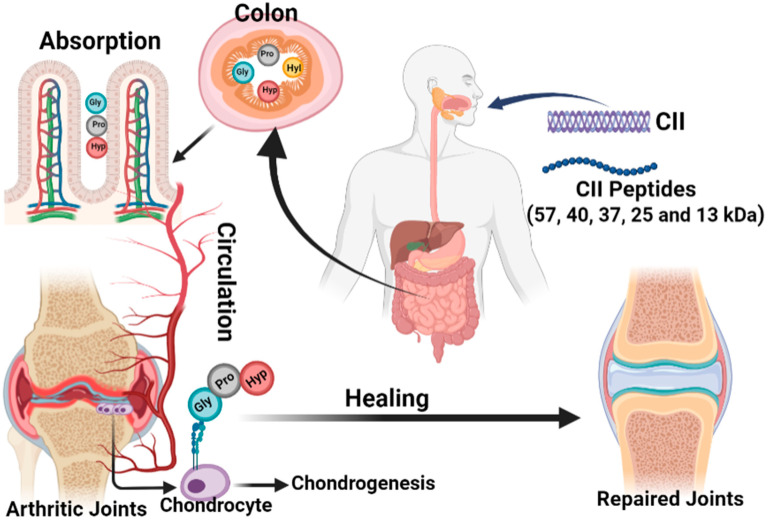
Schematic representation of the anti-arthritic mechanism of collagen and its peptides. Collagens with different molecular weights are obsorbed in the colon and are transported to the arthritic joints by circulation as tri-peptides for healing arthritis.

The anti-arthitic mechanism of collagen and its peptides is illustrated in Figure 3. The most abundant protein in articular cartilage and intervertebral discs is CII; that is why they are believed to support cartilage growth, repair, and healing without adverse effects through oral supplementation. Though several studies confirmed the arthritogenic mechanism of CII as described in Section 2, in contradiction, a similar amount of research also substantiates the anti-arthritogenic effect of collagens including type I and II. Our previous in vitro and in vivo studies also confirmed the immunosuppressive effect of CII peptides with 57, 40, 37, 25, and 13 kDa [112,113,114,115]. So in this section, we are exploring the molecular mechanism of collagen and its peptides on the anti-arthritogenic effect.

Table 1 summaries the studies on anti-arthritogenic effect of collagen and its peptides. Previously, Paul et al. reported that collagen supplements containing a rich amount of proline, glycine, and hydroxyproline improve the joint cartilage physiology and protect against oxidation and inflammation [116]. More surprisingly, strong evidence to support the protective effect of the CII supplement against joint swelling in RA was reported in Science by Trentham et al. [117].

One of the main intentions of oral administration of collagen supplements is related to oral tolerance to suppress or lower the immune response against orally administered antigens [118,119,120]. As discussed earlier, the tolerance response depends on the complete helix structure of collagen, since specific epitopes of intact helix structure interact with the immune system. It is believed that the lower molecular weights (MW) peptides can be readily absorbed (postprandial absorption) in the small intestine and transported through the peripheral circulation to the rest of the body including bones and joints, where it supports homeostasis [121,122]. In order to facilitate easy absorption and get desired peptide fragments (amino acid sequence) for better beneficial effects, collagen has been specifically hydrolyzed in several ways with enzymes and thermal denaturation to obtain collagen hydrolysates or peptides. Based on the type of process, vastly different collagen peptides with unique peptide sequences and MW can be obtained. Since the biological functions like regulating joint inflammation and subchondral bone of collagen peptides can potentially be affected by these factors. Besides, the lower MW collagen peptides (amino acid pool) can facilitate better absorption in the small intestine and thereby reach the target tissue including joints. Our previous studies claimed that the hydrolyzed collagen peptides with MW of 8–100 kDa were absorbed by intestinal villi and the absorption effect was more facilitated with 37 kDa through the small intestine [123,124]. Others reported that collagen peptides ranging from 1.5–2.5 kDa were potentially absorbed on the serosal side of the intestine and recovered in joints [125]. Further studies reported that the collagen peptides containing proline or hydroxyproline amino acid are resistant to the gastrointestinal digestive system and are thereby absorbed by the small intestine as intact peptides, which are potentially available in circulation for up to 4 h after ingestion [126,127]. The signals are initiated by binding collagen peptides with the α2A-domain of integrin receptor and contribute the chondrogenic growth [128,129].

Few reports claim that the collagen peptides possibly reduce systemic T cell attacks on the cartilage to avoid cartilage damage and joint inflammation through the interaction of epitopes with gut-associated lymphoid tissue [122,130,131]. In this case, the administered collagen hydrolysates were digested and further converted into smaller peptides in order to eliminate the immunomodulatory properties. Moreover, the smaller peptides increase the chance of bioavailability due to better absorption than undenatured collagen [121,132]. To support this finding, few in vitro studies claimed that collagen peptides can accrue in cartilage with high doses, upregulate chondrocytes to produce macromolecules of the extracellular matrix (ECM), and upregulate and downregulate the osteoblast and osteoclast activity, respectively [125,133,134,135,136]. Similarly, an earlier in vivo study also claims the same, where the orally administered collagen hydrolysate end products accumulate in cartilage after intestinal absorption and increase ECM macromolecules synthesis [133]. The above human trial study (four open-label and three double-blind) conducted by Bello and Oesser also claimed biocompatibility and protective effect of collagen against pain in men and women with arthritis and other disease conditions.

Collagen peptides with doses of 25 g/day and 50 g/day were orally administered to the horse with acute synovitis induced by intra-articular injection of 0.5 ng lipopolysaccharide (LPS) of Escherichia coli. Collagen peptides had an anti-inflammatory effect by reducing prostaglandin E2 than the placebo group. Surprisingly, the horses treated with collagen peptides improved movement and willingness to run than the placebo group [137,138].

The first clinical trial with a healthy population was conducted with 147 healthy athletes, who were given 25 mL of a liquid formulation (containing 10 g of collagen hydrolysates) and liquid xanthan (placebo group) showed that supplementation of collagen hydrolysates potentially improved joint discomfort and pain during walking, standing, carrying objects, and lifting [139]. In another clinical study, 15 healthy male subjects were given a single dose of an oral formulation of 10 g of collagen hydrolysate in 100 mL of milk or water. The study results evidenced the presence of a higher amount of collagen-specific amino acids (such as glycine, proline, hydroxyproline, and hydroxylysine) concentration in plasma and reach the tissues in the synovial joint [140]. A seminal study conducted with 250 primary knee arthritis subjects, who were given the same amount as reported earlier, i.e., 10 g collagen hydrolysates per day for 180 days, concluded that the collagen hydrolysates are safe as a functional food ingredient and potentially improve the knee joint complications in arthritis [141]. In another randomized double-blind pilot trial study, McAlindon et al. investigated the effect of collagen hydrolysates in osteoarthritic cartilage, using delayed gadolinium enhanced magnetic resonance imaging of cartilage (dGEMRIC) technique and reported a higher dGEMRIC score in the medial and lateral tibial regions of interest for collagen hydrolysate treated group compared to placebo groups [142].

**Table 1:** List of studies conducted on collagen and peptides related to arthritis.

Study Type	Type of Collagen and Molecular Weight	Year	Outcome	Reference
in vitro/in vivo	CII 37 KDa	2015	Antioxidant	[113]
in vitro	CII peptide	2019	Antioxidant	[114]
in vitro	CI peptide	2017	Immunologic tolerance	[115]
in vitro	CII (25, 40 and 57 KDa)	2016	Suppress immune response	[116]
in vivo	Collagen peptide	2019	Maintaining amino acid balance	[117]
Clinical trials	Collagen Hydrolyzates	2021	Support joint health and anti-osteoarthritis (OA)	[121]
Clinical trials	Collagen hydrolyzates	2019	Increases the postprandial plasma concentration of aminoacids	[122]
Clinical trials	CII	2022	Preventing joint inflammation of OA and RA	[131]
in vitro/in vivo	Collagen hydrolyzates	2010	Increasing osteoblastogenesis and improve bone metabolism	[136]
in vivo	CII hydrolyzates	2012	Improve OA related symptoms	[137]
in vivo	Collagen peptide	2018	Relief OA symptoms	[139]
Clinical trials	CII hydrolyzates	2008	Support joint health	[140]
Clinical trials	Collagen hydrolyzates	2011	Patient exhibited changes in proteoglycan content in knee	[143]

### 3.2. Negative Impact of Collagen in Arthritis

On contrary, few reports claimed insufficient evidence to put forward the potential practice of collagen hydrolysates in daily practice for the treatment of arthritis patients [143]. To support this finding, a study conducted with heterogeneous collagen hydrolysates (From porcine: Mobiforte^®^, Astrid Twardy GmbH, Unterföhring, Germany and fish: Peptan^®^ F 5000 and Peptan^®^ F 2000 from Rousselot SAS, Puteaux, France) showed a negative impact on modulating collagen biosynthesis in human knee cartilage explants, and there was also evidence of elevated aggrecanases (ADMATS4 and ADMATS5), IL-6, MMP-1, -3, and -13 levels [144]. We believe that, as described before, the collagen type-I/II may have a different pattern of amino acid composition and sequences based on the type of hydrolysis method, which may responsible for the contradictory outcome.

### 3.3. Molecular Mechanism of Collagen for Cartilage Homeostasis

None of the available literature provided strong evidence on the actual mechanism of collagen hydrolysates in the protective effect of arthritis throughout in vivo and clinical studies. However, several in vitro studies carried out to evaluate the mechanism of collagen in cartilage cells growth. For instance, fish skin collagen hydrolysates from haddock, pollock, cod, jellyfish, chub, blue shark, and tilapia were reported to induce chondrogenic differentiation from stem cells [145,146,147,148,149,150,151]. The available data from these in vitro studies explained the signaling mechanism of collagen peptides in chondrocytes’ growth. Fish collagen hydrolysates up-regulated the protein and gene expression of osteogenic markers (RUNX2, ALP, OPN, and OCN) and activated the mitogen-activated protein kinase (MAPK)/extracellular signal-regulated kinases (ERK) signaling pathway [152]. In general, the chondrogenic efficiency of collagen peptides is achieved by activating several signaling molecules such as Sox9, Indian hedgehog (Ihh), bone morphogenetic proteins (BMPs), parathyroid hormone-related peptide (PTHrP), β-catenin, and fibroblast growth factor receptor 3 (FGFR3) [153]. However, it is not possible to expect to get a similar outcome in in vitro studies (conducted under a controlled environment) same as in vivo studies, since several micro-environmental factors play role in in vivo condition. Therefore, convincing empirical evidence is necessary to strongly support the safety, inflammatory, and anti-arthritogenic aspects of collagen hydrolysates at both academic and industrial levels in order to develop collagen-based nutraceutical supplements for the treatment of arthritis.

## 4. Conclusions

Overall, we understand that collagen in its native form may induce arthritis and the level of arthritis induction highly depends on molecular structures and the presence of epitopes in the triple helix. More specifically, the effect of collagen in arthritis induction is mainly carried out by the involvement of either B cells or T cells. In both ways, the opposite cells are activated by the paracrine signaling mechanism of immune cells. On the other side, collagen hydrolysates or peptides possibly assuming without the arthritogenic epitopes could protect the cartilage from being damaged by the immune system by potentially minimizing immune response through tolerance. After all these assessments, we opined that the paradoxical dual role of CII in RA is purely dependent on the triple helix structures; amino acid sequence; major epitopes on triple-helical CII (256–270) such as C1, U1 and J1, amino acid residues (glycine, proline, hydroxyproline, and hydroxylysine); and the glycosylation pattern of CII. However, the big riddle is how our body does not recognize the CII in cartilages and CII degraded byproducts during collagen turnover as autoantigen to trigger arthritis in normal biological activity. The other riddle is how the immune system distinguishes the biologically available CII and external CII and processes them in two different ways. Therefore, much work, including in vitro, in vivo, and clinical trials, is needed to address the controversial hypothesis of collagen use in arthritis.

## Abbreviations

CI	Type I collagen
CII	Type II collagen
MMPs	Matrix metalloproteinases
NK cells	Natural Killer cells
DCs	Dendritic cells
CIA	Collagen-induced arthritis model
RA	Rheumatoid arthritis
OA	Osteoarthritis
SFs	Synovial fibroblasts
mAbs	Monoclonal antibodies
TCR	T cell receptor
LPS	Lipopolysaccharide
RUNX2	Runt-related transcription factor 2
ALP	Alkaline Phosphatase
OPN	Osteopontin
OCN	Osteocalcin
TNF-α	Tumor necrosis factor-α
IL	Interleukin
TGF	β- Transforming growth factor β
ECM	Extracellular matrix
MAPK	mitogen-activated protein kinase
ERK	extracellular signal-regulated kinases
Sox9	SRY-related high-mobility group-box gene 9
